# Hydrogen sulfide alleviates postharvest ripening and senescence of banana by antagonizing the effect of ethylene

**DOI:** 10.1371/journal.pone.0180113

**Published:** 2017-06-29

**Authors:** Yun Ge, Kang-Di Hu, Sha-Sha Wang, Lan-Ying Hu, Xiao-Yan Chen, Yan-Hong Li, Ying Yang, Feng Yang, Hua Zhang

**Affiliations:** 1 School of Food Science and Engineering, Hefei University of Technology, Hefei, China; 2 College of Environment and Energy Engineering, Anhui Jianzhu University, Hefei, China; 3 Xuzhou Institute of Agricultural Sciences of the Xuhuai District of Jiangsu Province, Xuzhou, China; Universidade do Minho, PORTUGAL

## Abstract

Accumulating evidence shows that hydrogen sulfide (H_2_S) acts as a multifunctional signaling molecule in plants, whereas the interaction between H_2_S and ethylene is still unclear. In the present study we investigated the role of H_2_S in ethylene-promoted banana ripening and senescence by the application of ethylene released from 1.0 g·L^−1^ ethephon solution or H_2_S with 1 mM sodium hydrosulfide (NaHS) as the donor or in combination. Fumigation with ethylene was found to accelerate banana ripening and H_2_S treatment effectively alleviated ethylene-induced banana peel yellowing and fruit softening in parallel with decreased activity of polygalacturonase (PG). Ethylene+H_2_S treatment also delayed the decreases in chlorophyll and total phenolics, and increased the accumulation of flavonoid, whereas decreased the contents of carotenoid, soluble protein in banana peel and reducing sugar in pulp compared with ethylene treatment alone. Besides, ethylene+H_2_S treatment suppressed the accumulation of superoxide radicals (·O_2_^−^), hydrogen peroxide (H_2_O_2_) and malondialdehyde (MDA) which accumulated highly in ethylene-treated banana peels. Furthermore H_2_S enhanced total antioxidant capacity in ethylene-treated banana peels with the 2,2’-azobis(3-ethylbenz-thiazoline-6-sulfonic acid (ABTS) assay. The result of quantitative real-time PCR showed that the combined treatment of ethylene with H_2_S down-regulated the expression of ethylene synthesis genes *MaACS1*, *MaACS2* and *MaACO1* and pectate lyase *MaPL* compared with ethylene treatment, while the expression of ethylene receptor genes *MaETR*, *MaERS1* and *MaERS2* was enhanced in combination treatment compared with ethylene alone. In all, it can be concluded that H_2_S alleviates banana fruit ripening and senescence by antagonizing the effect of ethylene through reduction of oxidative stress and inhibition of ethylene signaling pathway.

## Introduction

Banana (*Musa acuminata*, AAA group) is one of the most popular fruits in the world with high nutritional and economical values [1]. As a typical climacteric fruit, the ripening of banana fruit is initiated by an autocatalytic increase in ethylene biosynthesis, which produces a respiration peak [2]. The progress of banana ripening can be divided into both the changes in the pulp and in the peel of fruit, and the ripening process occurs outward normally from the inside of banana with pulp ripening preceding peel yellowing [3]. As the banana fruit is ripe, it has a very short shelf life due to the soft texture, peel browning and susceptibility to diseases, leading to severe postharvest loss. Besides the critical role of ethylene in banana ripening, oxidation injury caused by reactive oxygen species (ROS) plays important role in quality deterioration [4]. ROS is highly reactive and causes lipid peroxidation, which could result in undesirable flavors and odors due to increased LOX activity and malondialdehyde (MDA) content in fruit tissues [5]. Therefore, developing strategies to attenuate ethylene synthesis and inhibit ROS production could be effective for reducing quality deterioration and extending the storage life of banana fruit.

Hydrogen sulfide (H_2_S) is emerging as a new gaseous signaling molecule in diverse organisms such as bacteria, fungi, worms, humans, and plants [6]. In plants, H_2_S is synthesized via cysteine degradation catalyzed by D-/L-cysteine desulfhydrase (D-/L-CDes), or through reducing SO_3_^2-^ by sulfite reductase [7]. Besides, β-cyanoalanine synthase also catalyzes the generation of H_2_S and β-cyanoalanine during the detoxification of cyanide in mitochondria [8]. Accumulating evidence indicates that H_2_S is involved in various processes in plants, including seed germination, root organogenesis, abiotic stress tolerance, photosynthesis, guard cell movement and autophagy, suggesting that H_2_S acts as an important signaling molecule in plants, of comparable importance to NO and CO in mammalian systems [9–16]. Several recent studies found that H_2_S could alleviate postharvest senescence by reducing oxidative stress through modulating antioxidant enzymes in strawberry, mulberry, kiwifruit, and broccoli etc [5, 17–19].

Ethylene is one of the most important hormones in regulating almost every phase of plant growth and development [20]. The role of ethylene has been widely studied in climacteric fruit ripening which exhibits a burst in ethylene production including banana, mango, apple etc. Ethylene signal triggers several changes that lead to conversion of starch into free sugars, generation of aroma, degradation of chlorophyll, accumulation of carotenoids and degradation of cell wall component [21]. Various strategies have been developed to inhibit the effect of ethylene. For instance, 1-methylcyclopropene (1-MCP) is used to block ethylene action and to prolong fruit shelf-life [22].

Although the effects of H_2_S on alleviating ripening and senescence are confirmed in fruits, there is still a lack of information about the molecular interaction between H_2_S and ethylene signaling. A better understanding of the physiological basis involved in the role of H_2_S is of importance to elucidate the mechanisms of fruit ripening and senescence and develop strategies for banana quality control. In the present study, the combination of ethylene with H_2_S was applied to banana, and the effects of H_2_S signal on banana senescence, ROS metabolism and ethylene signaling were investigated, thereby providing evidence regarding the possible role of H_2_S in ethylene-promoted banana ripening and senescence.

## Materials and methods

### Plant materials and treatment

Hands of mature-green bananas (*Musa* spp., AAA group cv. ‘Brazil’) were harvested at a commercially mature stage (70–80%) from a commercial orchard named Tian Tian Farm in Hainan, China and were immediately delivered to the laboratory. Upon arrival, banana hands were separated into fingers and selected for uniformity of size, color and absence of damage and disease. 1.0 g·L^–1^ ethephon solution (2-chloroethylphosphonic acid, resolved in phosphate buffer (pH7.5) which was found to effectively promote maturation of banana was used as an ethylene donor. 1 mM sodium hydrosulfide (NaHS) solution which released a stable level of 1.50×10^−10^ mol·L^–1^ H_2_S was used to release H_2_S. The selected fingers were randomly divided into four groups of 16 fingers (280 g ± 10 g) for each treatment. Fingers of the first group i.e. control group were stored in sealed containers (volume 3 L) at 25°C with a relative humidity of 85–90%. Fingers of the second group (ETH group) in the container were fumigated with ethylene released from 100 mL of 1.0 g·L^–1^ ethephon solution. The third banana group (H_2_S group) in the container was fumigated with H_2_S released from 150 mL 1 mM NaHS. The fourth banana group (ETH+H_2_S group) was stored in a container containing 150 mL of 1 mM NaHS and 100 mL of 1.0 g·L^–1^ ethephon solutions which were stored in two separate beakers. The solutions were renewed daily and the bananas were photographed daily for 6 days. Banana peel or pulp in the middle region of bananas were sampled on Day 0, 1, 3, 5 of storage and subsequently frozen in liquid nitrogen and stored at –80°C for further analysis. All samples were prepared with three biological replicates.

### Measurement of banana color

The change in banana peel color was measured by colorimeter (model WSC-100; Konica Minolta, Tokyo, Japan), which reads the values of L*, a* and b*. L* stands for lightness, a* shows chromaticity on a green (–) to red (+) axis, and b* chromaticity on a blue (–) to yellow (+) axis. Each banana was measured at 6 equidistant points around the middle area on a banana finger.

### Fruit firmness evaluation

Banana firmness was measured using a texture analyzer (Model TA.XT plus, SMS) around the middle area of each banana. The cross-head speed was 5 mm·s^–1^ and the penetration depth was 5 mm. Fruit firmness values were an average of 6 measured values of each banana ± SD (standard deviation).

### Activity assay of polygalacturonase

Polygalacturonase (PG, EC 3.2.1.15) activity was assayed by the method of Pathak and Sanwal [23]. Banana pulp (1.0 g) was homogenized with 4 mL ice-cold acetate buffer and peels (1.0 g) with 6 mL buffer. The homogenate was centrifuged at 10,000 *g* at 4°C for 30 min. Then the supernatant was used for PG activity assay. The analysis was repeated three times for each treatment. PG activity was expressed as U·g^–1^ FW (fresh weight).

### Determination of chlorophyll and carotenoid contents

The contents of chlorophyll and carotenoid of banana peels were determined according to the method of Lichtenthaler and Wellburm [24] and Nath et al. [25] respectively. Each analysis was repeated three times and the results chlorophyll and carotenoid were expressed as mg·g^–1^ FW (fresh weight).

### Determination of the contents of total phenolics, flavonoids, soluble protein and reducing sugar

Determination of total phenolics and flavonoids in banana peels was performed according to the methods of Pirie and Mullins [26] and Zhishen et al. [27], respectively.

Soluble protein content was measured according to the method of Bradford [28]. Banana peels (1.00 ± 0.05 g) were ground in 5 mL of phosphate buffer (pH7.0, 200 mM). Analyses were repeated in triplicate and the results expressed as mg·g^–1^ FW. Reducing sugar content was measured according to Mille [29]. Banana pulps (1.0 g) were homogenized in 4 mL ice-cold phosphate buffer. After centrifugation, the supernatant (0.2 mL) was mixed with 1.5 mL of 3,5- dinitrosalicylic acid and 1.8 mL of dH_2_O, then the mixture was heated at 100°C for 5 min, cooled, and added to 25 mL distilled water. Reducing sugar was determined at 540 nm by a spectrophotometer, and the results were expressed as mg·g^–1^ FW (fresh weight).

### Determination of superoxide anion (·O_2_^−^), hydrogen peroxide (H_2_O_2_) and malondialdehyde (MDA) in banana peel

Contents of ·O_2_^*−*^, H_2_O_2_ and MDA were determined according to the methods described by Hu et al. [17] with slight modifications. The generation rate of ·O_2_^*−*^ was determined using hydroxylamine method. Banana peel samples (0.50 ± 0.05 g) were ground with 3 mL of 50 mM Tris-HCl buffer (pH7.8) and the homogenate was centrifuged at 12,000 *g* at 4°C for 30 min. The reaction mixture (0.5 mL) contained 50 mM Tris-HCl buffer (pH7.5), 0.5 mM XTT [sodium, 3-1- (phenylamino-carbonyl)-3, 4-tetrazolium-bis(4-methoxy-6- nitro), and benzenesulfonic acid hydrate], and 50 μL of sample extracts. Corrections were made for the background absorbance in the presence of 50 U of superoxide dismutase (SOD). The rate of ·O_2_^*−*^ production was expressed as μg·g^−1^ FW (fresh weight) · s^−1^.

For determination of H_2_O_2_, banana peels (0.50 ± 0.05 g) were ground and extracted in 3 mL cold acetone. The homogenate was centrifuged at 10,000 *g* at 4°C for 30 min and 0.5 mL of the supernatant fraction was mixed with 1.5 mL of CHCl_3_ and CCl_4_ (1:3, V/V) mixture, then 2.5 mL of distilled water was added and the mixture centrifuged at 10,000 *g* for 1 min and the aqueous phase collected for H_2_O_2_ determination. The reaction system included 0.5 mL aqueous phase, 0.5 mL of buffer (phosphate-buffered saline, 200 mM, pH7.8), and 20 μL (0.5 unit) of catalase as control or inactive catalase protein (catalase inactivated by heating in boiling water for 5 min). After incubation at 37°C for 10 min, 0.5 mL of 200 mM titanium 4-(2-pyridylazo) resorcinol (Ti-PAR) was added to the reaction mixture for further incubation at 45°C for another 20 min. Absorbance at 508 nm was measured and H_2_O_2_ content was indicated as μmol·g^−1^ FW (fresh weight).

For MDA analysis banana peel samples (0.50 ± 0.05 g) were ground in 3 mL of 0.1% trichloroacetic acid (TCA) and centrifuged at 10,000 *g* for 30 min, and 1.8 mL of the supernatant fraction was mixed with 1.8 mL of 20% TCA containing 0.5% thiobarbituric acid. The mixture was heated at 100°C for 30 min, cooled, and centrifuged at 15,000 *g* for 10 min. Absorbance was recorded at 532 nm and the value for nonspecific absorption at 600 nm was subtracted. An extinction coefficient of 155 mM^−1^·cm^−1^ was used to calculate MDA content and the content of MDA in banana peels was expressed as μmol·g^−1^ FW (fresh weight).

### Total antioxidant capacity measurement

To estimate the total antioxidant capacity in banana peels, the 2,2’-azobis(3-ethylbenz-thiazoline-6-sulfonic acid) (ABTS) free radical scavenging activity was determined according to the method described by Re et al. [30]. For ABTS assay, 0.15 g of frozen banana peel tissue was extracted with 80% methanol using a pre-chilled mortar and a pestle on an ice bath. The homogenate was filtered through two layers of muslin cloth and centrifuged for 15 min at 5000 *g* at 4°C and 10 μL sample extract was used for ABTS assays. 7 mM ABTS solution was prepared 16 h in advance, stored in the dark and then adjusted with methanol to an absorbance of 0.7 at 734 nm. Then an aliquot of 10 μL extract was added to 1 mL of ABTS solution, and measurements at 734 nm were registered each minute for five minutes. Radical scavenging capacity was expressed as the inhibition percentage and was calculated using the formula % radical scavenging activity = (control optical density − sample optical density/control optical density) × 100 as described in Girennavar et al. [31].

### Quantitative real-time PCR Analysis

Banana peel samples (0.15 g) were homogenized in liquid nitrogen, and total RNA was extracted by TRNzol RNA Reagent kit (Tiangen) according to the manufacturer’s instructions. The total RNA was used for cDNA synthesis using a reverse transcription kit (PrimeScript RT Master Mix, Takara, Kyoto, Japan). Total RNA from five treatments is used for first strand cDNA synthesis in a 20 μL reaction volume containing 4 μL 5 × PrimeScript RT Master Mix. Quantitative PCR is performed using a iQ^™^5 Real-Time PCR System with SYBR Premix Ex Taq (Takara, Kyoto, Japan) according to the manufacturer's instructions. PCR procedures were as follows: initial denaturation at 95°C for 5 min, followed by 40 cycles of 95°C for 30 s, 55°C for 30 s, and 72°C for 30 s. Primers used for quantitative PCR are shown in Table 1. *MaActin* was used as the reference gene. Relative quantification was processed using the method of Delta-Ct.

**Table 1:** Primers used for quantitative PCR.

Primer	sequence
	First line: forward/ second line: reverse
*MaACS1*	5'-GGGTCTCAGAGTTCAAAGCA-3'
(GenBank: AB021906.1)	5'-AGGATCAGCCAGGCAAAA-3'
*MaACS2*	5'-GGGAGAAGTAAGAGGAAACA-3'
(GenBank: AB021907.1)	5'-GAACAATGGATGGGAACG-3'
*MaACO1*	5'-CGAGTTCGCCAACAAAGC-3'
(GenBank: AJ223232.1)	5'-GGTCGTCCTGGAAGAGCA-3'
*MaACO2*	5'-TTCTGCGGGTCCAAAGGT-3'
(GenBank: X95599.1)	5'-TGACGACGATGGAGTGGC-3'
*MaETR*	5'-TATTTAGCACGACAGGAG-3'
(GenBank: AF113748.1)	5'-TGATTAGAGTTGCCAGAA-3'
*MaERS1*	5'-AGAATAAGGCAGAGGAAC-3'
(GenBank: AB266315.1)	5'-AATAATGGAACTCGGACA-3'
*MaERS2*	5'-GCTATGCGGTAATGGTTT-3'
(GenBank: AB266316.1)	5'-ATTCTGCCTCCTGTCGTG-3'
*MaPL*	5'-TGGAACTGGAGGTCGGAAGG-3'
(GenBank: X92943.1)	5'-CAGGCAGAGGTTGGTATCGTAATG-3'
*MaActin*	5'-GCTTACGTGGCACTTGACTA-3'
(GenBank: AB022041.1)	5'-TACCTGCTGACTCCATACCAAT-3'

### Statistical analysis

Statistical significance was tested by one-way analysis of variance (ANOVA) using IBM SPSS Statistics (SPSS version 20.0; Armonk, NY), and the results were expressed as the means ± SD (standard deviation).

## Results

### Effect of ethylene and H_2_S on the ripening and senescence of banana fruit

**Fig 1:**
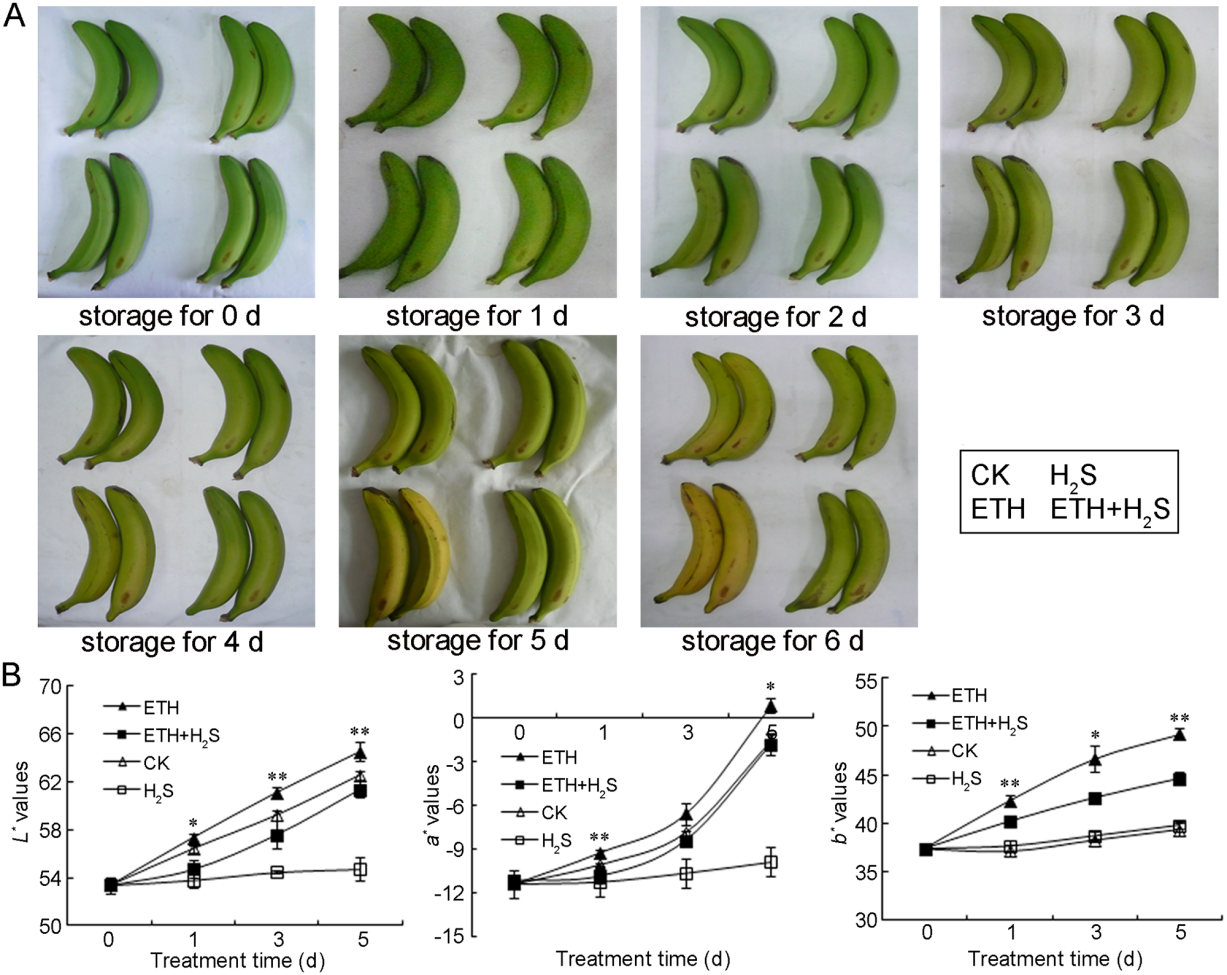
Effects of H_2_S and ethylene treatment on postharvest ripening (A) and chromatism values of L*, a*, b* of bananas (B). Banana fingers were respectively fumigated with water, 1.0 mM solution of H_2_S donor NaHS, 1.0 g·L^–1^ ethylene donor ethephon solution or 1.0 mM aqueous solution of NaHS plus 1.0 g•L^–1^ ethephon solution for 0–6 d at 25°C as shown in lower right part of A. Photographs (A) were taken from Day 0 to Day 6, and chromatism values of L*, a* and b* (B) of banana were recorded on 0, 1, 3 and 5 d. The symbols * and ** in this figure stand for significant difference between ETH and ETH+H_2_S at P<0.05 and P<0.01, respectively. CK: control group; H_2_S: H_2_S treatment; ETH: ethylene treatment; ETH+H_2_S: ethylene plus H_2_S treatment.

Banana fingers were fumigated with ethylene, H_2_S or ethylene+H_2_S to study the possible role of H_2_S in ethylene-induced fruit ripening and senescence. Our preliminary experiment indicated that 1.0 g·L^–1^ ethylene donor ethephon solution promoted banana ripening effectively. H_2_S donor NaHS solutions at 0.5 mM induced minor changes in banana peel color, while 1.0 mM of NaHS was found to alleviate the ripening of banana significantly (data not shown). Therefore, 1.0 g·L^–1^ ethephon solution or 1.0 mM aqueous solution of NaHS or in combination was applied to postharvest banana. As shown in Fig 1A, banana fruit fumigated with ethylene turned yellow after 3 days of storage, while H_2_S or ethylene+H_2_S treatments sustained greenness even after 4 days of storage and became yellow after 5 days. The change in banana peel color was evaluated in Fig 1B. Ethylene treatment induced more severe increase in L* value in banana fruit compare with control, whereas ethylene+H_2_S significantly attenuated the increase in peel lightness. H_2_S treatment alone tended to retain the lowest L* value and only minor increase was found during banana storage. The value of a* indicated chromaticity on a green (−) to red (+) axis, and the results showed that dramatic increase in a* value was observed in ethylene-treated banana fruit in comparison with the lower value in control or ethylene+H_2_S treatment. H_2_S treatment was found to maintain a* value at a relative stable level, suggesting that H_2_S treatment effectively retained greenness in banana peels. Ethylene treatment also induced an increase in b* value in banana fruit while ethylene+H_2_S significantly attenuated the increase (Fig 1B).

**Fig 2:**
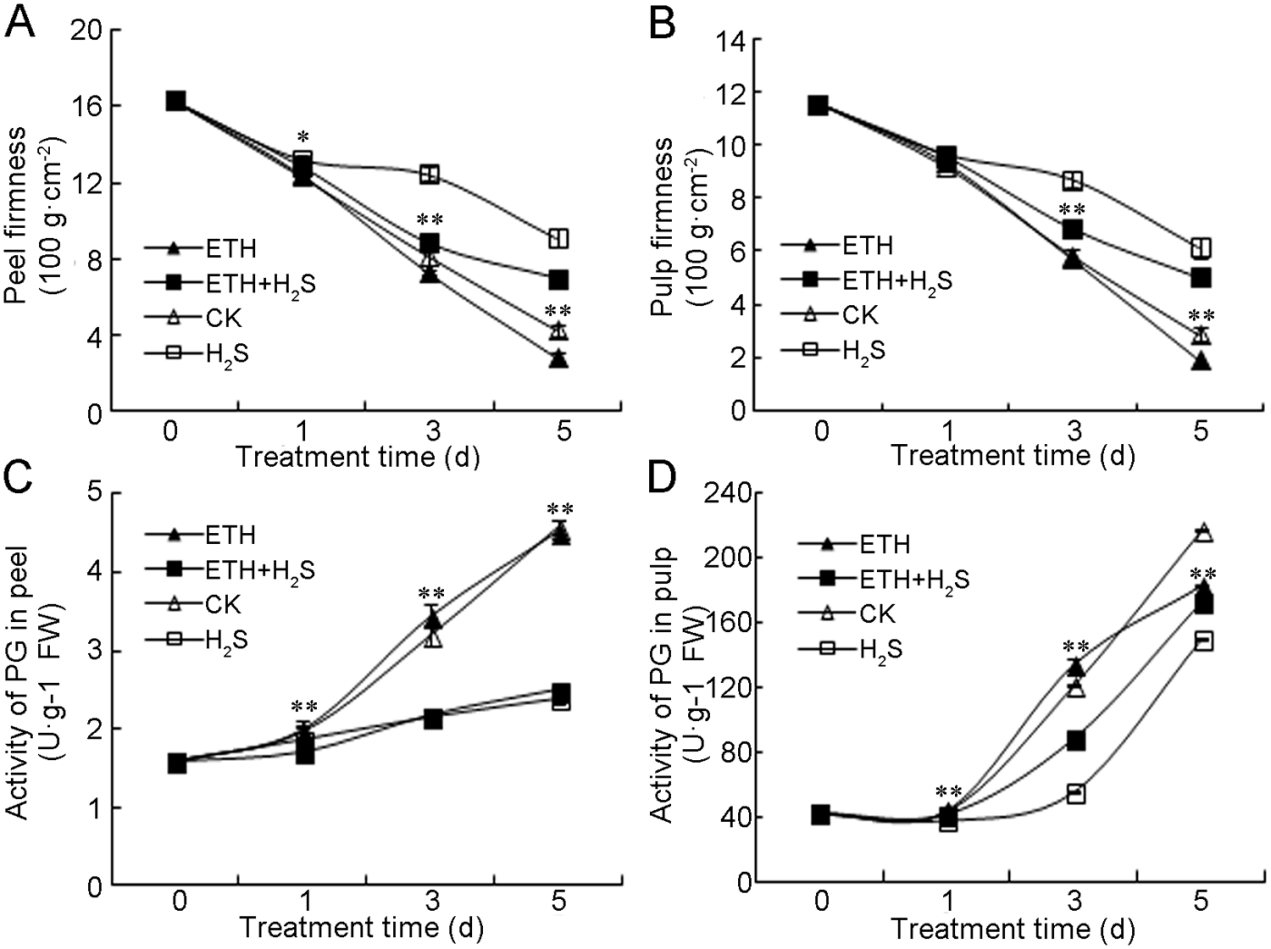
Effects of H_2_S and ethylene treatment on the firmness of banana of peel (A) and pulp (B) and on the activities of polygalacturonase (PG) in banana peel (C) and pulp (D). Bananas were respectively fumigated with water, 1.0 mM aqueous solution of NaHS, 1.0 g·L^–1^ ethephon solution or 1.0 mM aqueous solution of NaHS plus 1.0 g·L^–1^ ethephon solution for 0–5 d at 25°C. Data were recorded on 0, 1, 3 and 5 d and expressed as means ± SD of three independent experiments with three replicates. The symbols * and ** in this figure stand for significant difference between ETH and ETH+H_2_S at P<0.05 and P<0.01, respectively. FW: fresh weight. CK: control group; H_2_S: H_2_S treatment; ETH: ethylene treatment; ETH+H_2_S: ethylene plus H_2_S treatment.

During ripening, banana fruit undergoes softening due to ethylene-regulated cell wall degradation. Banana peel and pulp under control conditions softened during storage. Ethylene accelerated peel and pulp softening compared with control, while ethylene+H_2_S significantly inhibited this fruit softening (Fig 2A and 2B). In contrast, H_2_S treatment sustained highest firmness after 3 days of storage compared with the other three conditions (Fig 2A and 2B). PG plays an important role in cell wall degradation. PG activity increased significantly in control and ethylene-treated banana peels, while ethylene+H_2_S and H_2_S alone attenuated the increase in enzyme activity (Fig 2C). As shown in Fig 2D, PG activity increased dramatically in control and ethylene treated pulp after 1 day of storage, whereas the combination treatment of ethylene+H_2_S delayed the increase in the enzyme activity. Lowest PG activity was found in H_2_S alone treatment since 3 days of storage (Fig 2D).

### Effect of ethylene and H_2_S, alone and in combination, on the contents of chlorophyll, carotenoid, total phenolics, flavonoids, soluble protein in banana peel and reducing sugar in pulp

**Fig 3:**
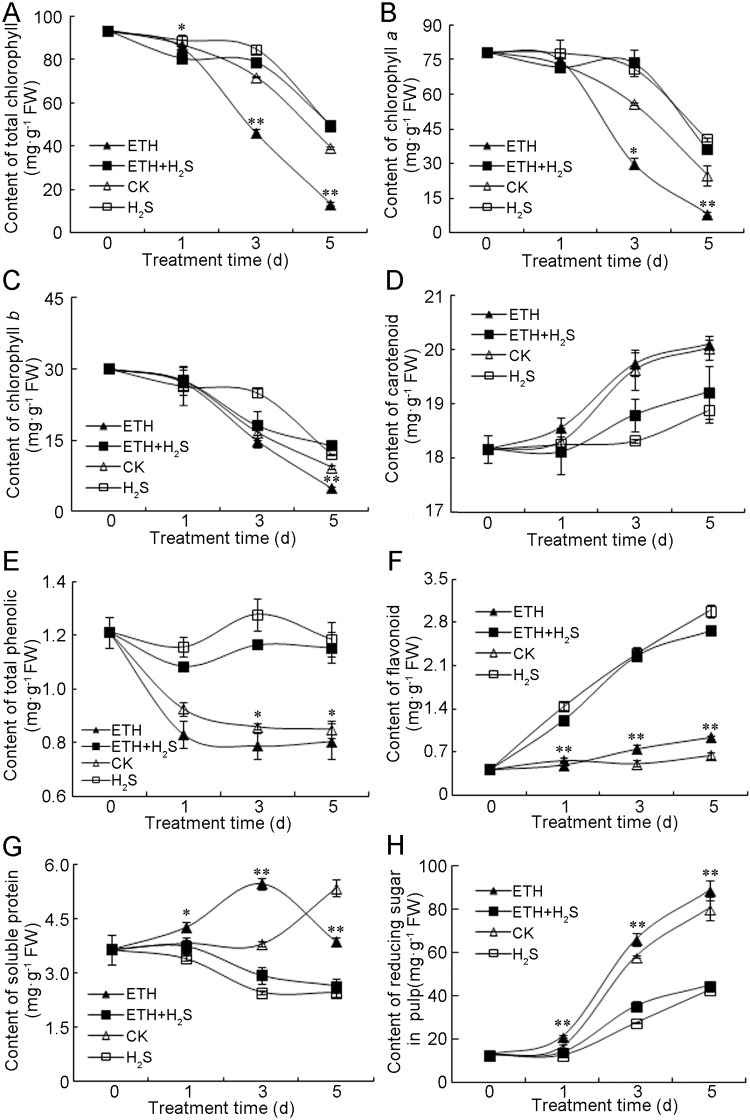
Effects of H_2_S and ethylene treatment on the contents of total chlorophyll (A), chlorophyll *a* (B), chlorophyll *b* (C), carotenoid (D), total phenolics (E), flavonoid (F), soluble protein (G) in banana peel and reducing sugar (H) in banana pulp. Bananas were respectively fumigated with water, 1.0 mM aqueous solution of NaHS, 1.0 g·L^–1^ ethephon solution or 1.0 mM aqueous solution of NaHS plus 1.0 g·L^–1^ ethephon solution for 0–5 d at 25°C. Data were recorded on 0, 1, 3 and 5 d and expressed as means ± SD of three independent experiments with three replicates. The symbols * and ** in this figure stand for significant difference between ETH and ETH+H_2_S at P<0.05 and P<0.01, respectively. FW: fresh weight. CK: control group; H_2_S: H_2_S treatment; ETH: ethylene treatment; ETH+H_2_S: ethylene plus H_2_S treatment.

During banana fruit ripening, the golden yellow color is due to chlorophyll (Chl) breakdown, which unmasks carotenoid pigments in the plastids [32]. As shown in Fig 3A, total chlorophyll in control banana peel decreased consistently, and ethylene accelerated this decrease. However, the combination treatment of ethylene+H_2_S or H_2_S alone prevented the loss of total chlorophyll. Chlorophyll *a* and *b* in the peels of four different conditions exhibited similar changes as total chlorophyll (Fig 3B and 3C). The content of carotenoid accumulated rapidly in peels of control and ethylene treatment (Fig 3D). On the contrary, the combination of ethylene+H_2_S or H_2_S alone significantly attenuated the increase in carotenoid content (Fig 3D).

Phenolics are important antioxidants to maintain the radical scavenging activity and thus constitute the non-enzymatic antioxidant system in plant tissues [33]. Fig 3E showed that the content of total phenolics in control and ethylene treated peels decreased sharply on Day 1 followed by a plateau since Day 3. However phenolics content in peels of the combination of ethylene+H_2_S or H_2_S alone fluctuated during storage and was significantly higher than that of control and ethylene treatment after 1 day of storage (Fig 3E). The change of flavonoids is shown in Fig 3F. Flavonoid content in control and ethylene-treated banana peels only showed minor increase during storage, whereas the content increased greatly in peel of ethylene+H_2_S and H_2_S treatment (Fig 3F).

Soluble protein content in control peels remained stable followed by an increase on Day 5, whereas ethylene treatment induced a gradual increase during the first 3 days of storage followed by a drop on Day 5 (Fig 3G). Ethylene+H_2_S and H_2_S treatment showed a gradual decline in soluble protein content and the content was significantly lower than those of control and ethylene treatment since Day 3 (Fig 3G). As shown in Fig 3H, reducing sugar content in banana pulp of all four conditions accumulated gradually along with fruit storage. However ethylene was found to accelerate the accumulation of reducing sugar in banana pulp, and H_2_S treatment was able to delay this accumulation (Fig 3H).

### Effect of ethylene and H_2_S, alone and in combination, on superoxide anion, H_2_O_2_ and MDA contents in banana peel

**Fig 4:**
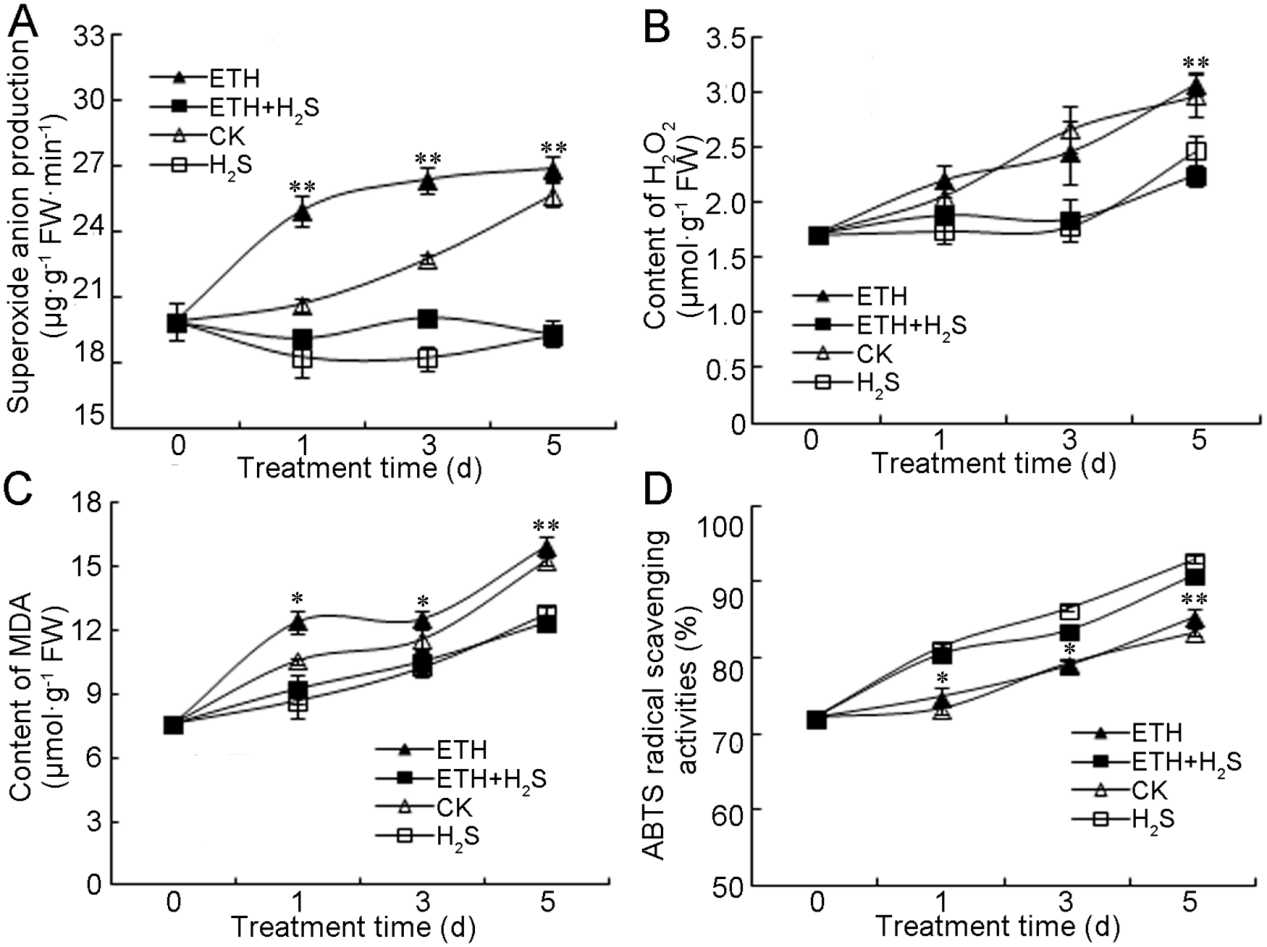
Effects of H_2_S and ethylene treatment on the production rate of superoxide anion (·O2−) (A), and contents of hydrogen peroxide (H_2_O_2_) (B), and malondialdehyde (MDA) (C) and ABTS radical scavenging activities (D) in banana peel. Bananas were respectively fumigated with water, 1.0 mM aqueous solution of NaHS, 1.0 g·L^–1^ ethephon solution or 1.0 mM aqueous solution of NaHS plus 1.0 g·L^–1^ ethephon solution for 0–5 d at 25°C. Data were recorded on 0, 1, 3 and 5 d and expressed as means ± SD of three independent experiments with three replicates. The symbols * and ** in this figure stand for significant difference between ETH and ETH+H_2_S at P<0.05 and P<0.01, respectively. FW: fresh weight. CK: control group; H_2_S: H_2_S treatment; ETH: ethylene treatment; ETH+H_2_S: ethylene plus H_2_S treatment.

Fruit senescence has been reported to be initiated by reactive oxygen species (ROS) [34]. To understand the antagonistic role of H_2_S in ethylene-induced fruit ripening and senescence, the contents of ROS and MDA, which is an index of lipid peroxidation, were determined in banana peels. As shown in Fig 4A, the generation of ·O_2_^−^ in control group showed an upward trend in the 5 days of storage, and ethylene treatment induced a more rapid increase in ·O_2_^−^ generation rate compared with control. On the contrary, ·O_2_^−^ generation was repressed significantly in ethylene+H_2_S and H_2_S treatment (Fig 4A).

As indicated in Fig 4B, H_2_O_2_ content in banana peel showed a consistent increase both in control and ethylene treatment, which increased nearly 1-fold after 5 days of storage. By contrast, H_2_O_2_ content in both ethylene+H_2_S and H_2_S treatments remained stable during the first 3 days of storage followed by an increase on Day 5, and was significantly lower than that of control and ethylene treatment (Fig 4B). MDA content showed a quick increase during the entire storage in all treatments as shown in Fig 4C, whereas the content in ethylene+H_2_S-treated banana peel was maintained at a significantly lower level compared with that of ethylene treatment. To evaluate the possible relationship between total antioxidant capacity and banana ripening and senescence, ABTS method was used to quantify the ability of the fruit to avoid oxidative damage. As indicated in Fig 4D, the free radical scavenging activity determined by ABTS in banana peels increased continuously during banana storage, and the values in ethylene+H_2_S and H_2_S conditions were significant higher than those in the control and ethylene treatment from Day 1 to Day 5.

### Effect of H_2_S and ethylene on the expression of genes in ethylene synthesis and signaling pathway and pectate lyase in banana peel

**Fig 5:**
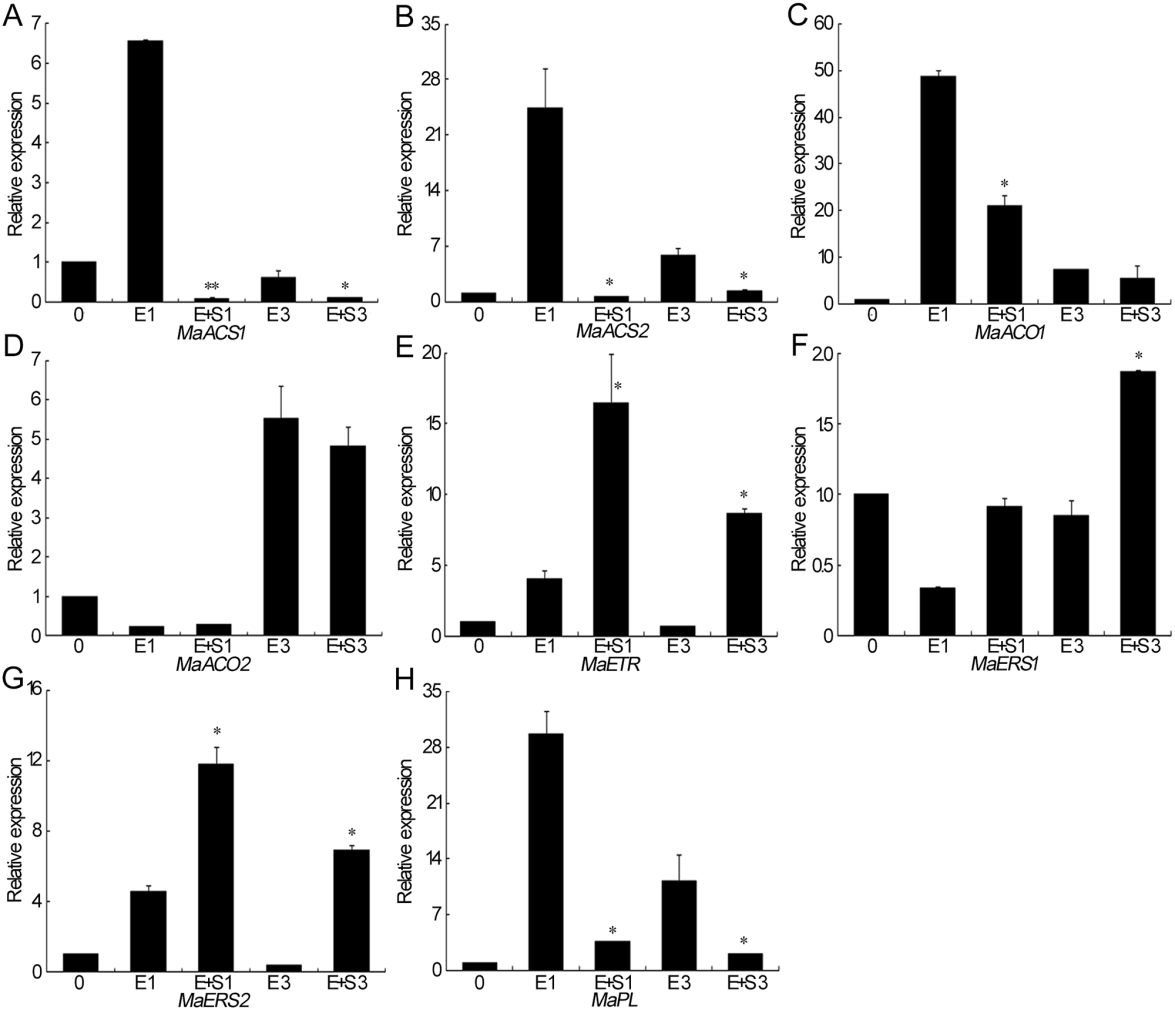
Effects of H_2_S and ethylene treatment on the relative gene expression of *MaACS1* (A), *MaACS2* (B), *MaACO1* (C), *MaACO2* (D), *MaETR* (E), *MaERS1* (F), *MaERS2* (G) *and MaPL* (pectin lyase gene) (H) in banana peel. Peels of bananas fumigated with 1.0 g·L^–1^ ethephon solution or 1.0 mM aqueous solutions of NaHS plus 1.0 g·L^–1^ ethephon solution were sampled on Days 0, 1 and 3 at 25°C. The symbols * and ** in this figure stand for significant difference between E and E+S at P<0.05 and P<0.01, respectively. Data were expressed as means ± SD of three independent experiments with three replicates. E: ethylene treatment; E+S: ethylene and H_2_S treatment.

In order to understand the role of H_2_S in ethylene signaling, the relative expressions of ethylene synthesis genes ACC synthase gene *MaACS1*, *MaACS2*, ACC oxidase *MaACO1*, *MaACO2* and ethylene receptor genes *MaETR*, *MaERS1* and *MaERS2*, and pectate lyase gene *MaPL* were analyzed in banana peel. Quantitative real-time PCR showed that the gene expressions of *MaACS1* and *MaACS2* were greatly induced by ethylene treatment on Day 1 and then decreased on Day 3, whereas H_2_S significantly repressed this induction in ethylene treatment (Fig 5A and 5B). Expression of *MaACO1* was up-regulated by ethylene and ethylene+H_2_S treatment, but the expression level in ethylene+H_2_S-treated peel was significantly lower than that of ethylene treatment (Fig 5C). Then expression of *MaACO1* decreased on Day 3 in both ethylene and ethylene+H_2_S treatment (Fig 5C). Fig 5D showed that a decrease was observed in gene expression of *MaACO2* on Day 1 followed by a dramatic enhancement in ethylene and ethylene+H_2_S-treated banana peels on Day 3.

Within the ethylene receptor family, *MaETR* expression was strongly induced by 16-fold in ethylene+H_2_S-treated banana peel but 4 fold in ethylene treatment (Fig 5E). Then the gene expression of *MaETR* was down-regulated on Day 3 in both treatments, but the expression level in ethylene+H_2_S treatment was significantly higher than that of ethylene. Similarly, ethylene+H_2_S treatment induced higher gene expression of two other ethylene receptor genes *MaERS1* and *MaERS2* compared with ethylene treatment (Fig 5F and 5G). Fig 5H showed that the expression of pectate lyase gene *MaPL* was greatly up-regulated in peels after 1 day of ethylene treatment followed by a decrease, while the expression in ethylene+H_2_S treatment underwent fewer changes. Statistical analysis indicated that *MaPL* expression in ethylene+H_2_S treatment was significantly lower than that in ethylene treated peels (Fig 5H).

## Discussion

Banana fruit after harvest undergo rapid ripening and senescence associated with loss of nutrients, peel browning and softening, resulting in a short shelf life [35]. The reduction of fruit spoilage and the enhancement of storability to deliver benefits to consumers are the most important goal for fruit dealers. As a typical climacteric fruit, banana ripening is initiated with a sharp increase in ethylene production. 1-methylcyclopropene (1-MCP) is a cyclic alkene that is able to block ethylene action and had a significant role in prolonging the fruit shelf-life [22]. However, banana fruit treated with 1-MCP may stay green or ripen with an uneven color [22], which limits the commercial application of 1-MCP in banana and highlights the need for alternative strategies to attenuate the role of ethylene in ripening [36]. In the present study, we found that ethylene released from 1.0 g·L^–1^ ethephon solution accelerated banana ripening and H_2_S with 1 mM sodium hydrosulfide (NaHS) as the donor effectively alleviated ethylene-induced banana peel yellowing and fruit softening. The relative stable content of chlorophyll under ethylene+H_2_S fumigation compared with ethylene alone, as shown in Fig 2A, strongly supports the role of H_2_S in preventing chlorophyll degradation. Meanwhile the combination treatment decreased the accumulation of carotenoid in banana peel and reducing sugar in pulp compared with ethylene treatment alone. Consistently, Ammawath et al. [37] also reported that banana peel turns from stage “green” to stage “trace of yellow” accompanied with the synthesis of new pigments such as carotenoid and the breakdown of the green pigment chlorophyll. During the ripening of bananas, the most important chemical changes are the hydrolysis of starch and the accumulation of sugars with parallel increases in sweetness of the pulp [38], which explained the lower level of reducing sugar in ethylene+H_2_S treated banana pulp compared with ethylene treated ones.

Excessive reactive oxygen species (ROS) and oxidative damage are responsible for fruit senescence and deterioration [34]. Plant cells have developed complex antioxidant enzymatic and non-enzymatic systems to cope with excessive ROS generation [39]. Non-enzymatic antioxidants such as phenolics and flavonoid, which are important quality attributes, also help to maintain a balanced ROS metabolism by quenching ROS [40]. In the present study, ethylene+H_2_S treatment was found to delay the decrease in total phenolics, and increased the accumulation of flavonoid compared with ethylene alone, highlighting the protective role of H_2_S in banana storage. Analysis of ROS such as ·O_2_^−^ and H_2_O_2_ revealed that ethylene+H_2_S treatment suppressed ROS accumulation. Besides, lipid peroxidation, which was evaluated by MDA content, is one of the most widely used indicators of membrane damage. As shown in Fig 4C, H_2_S significantly attenuated the accumulation of MDA in ethylene-treated banana peels during storage. Further, total antioxidant capacity was determined by ABTS method, which indicating that ethylene+H_2_S induced higher free radical scavenging activity than ethylene alone. These results indicate that H_2_S could alleviate ethylene-caused postharvest senescence of banana by reducing oxidative damages.

Ethylene plays a crucial role in the ripening and senescence of banana. Ethylene is synthesized from methionine via S-adenosylmethionine and 1-aminocyclopropane-1-carboxylic acid (ACC). Two key enzymes in this pathway are ACC synthase (converting S-adenosylmethionine to ACC) and ACC oxidase (converting ACC to ethylene) [41], which also been identified in banana fruit [2]. To understand the effect of H_2_S on ethylene pathway, we assayed the expression patterns of genes that are involved in ethylene biosynthesis (*MaACS1*, *MaACS2*, *MaACO1* and *MaACO2*) and signal transduction (*MaETR*, *MaERS1*, *MaERS2*) in banana peels. Ethylene treatment enhanced the expression of *MaACS1*, *MaACS2* and *MaACO1*, while the enhancement was greatly attenuated in the combined treatment of ethylene with H_2_S. However, ethylene+H_2_S enhanced the expression of ethylene receptor genes *MaETR*, *MaERS1* and *MaERS2* compared with ethylene alone. Ethylene receptors act as negative regulators which actively repress expression of ethylene responsive genes in the absence of ethylene and are inactivated by ethylene binding [42–43]. Thus the higher expression of *MaETR*, *MaERS1* and *MaERS2* observed in ethylene+H_2_S treatment might help to inhibit the effect of ethylene. Fruit softening is associated with cell wall degradation by cell wall hydrolases such as polygalacturonase (PG), pectin methyl esterase (PME), pectate lyase (PL) and cellulose [44]. And ethylene can promote the activities of these hydrolases during fruit ripening [45–46]. In the present study, ethylene treatment enhanced PG activity, while the combination of ethylene with H_2_S attenuated this enhancement. The pectate lyase (PL) showed an increase in transcript level during banana ripening [45]. Consistently Fig 5H showed that the expression of pectate lyase gene *MaPL* was greatly up-regulated in peels after ethylene treatment, while the expression in ethylene+H_2_S treatment was repressed, all implying that H_2_S may play an antagonizing role in the pathway of ethylene.

In conclusion, our results indicated that H_2_S could alleviate postharvest senescence of banana and maintain high fruit quality by decreasing ROS accumulation, improving natural antioxidant contents and radical scavenging capacity, and reducing lipid peroxidation, thereby maintaining the stability of the membrane structure. Besides, we provided strong evidence that H_2_S may play an antagonizing role in the pathway of ethylene by inhibiting ethylene biosynthesis and signaling pathway.
