# Correction: Hydrogen sulfide alleviates postharvest ripening and senescence of banana by antagonizing the effect of ethylene

**DOI:** 10.1371/journal.pone.0191351

**Published:** 2018-01-11

**Authors:** Yun Ge, Kang-Di Hu, Sha-Sha Wang, Lan-Ying Hu, Xiao-Yan Chen, Yan-Hong Li, Ying Yang, Feng Yang, Hua Zhang

The Funding section contains incomplete information. The complete funding information is as follows: This work was supported by National Natural Science Foundation of China (31670278, 31470013 to HZ; 31300133 to KH), Anhui Provincial Science and Technology Major Project (16030701073 to HZ) and Anhui Provincial Education Department (KJ2015ZD12 to YY). The funders had no role in study design, data collection and analysis, decision to publish, or preparation of the manuscript.

## References

[1] Ge Y, Hu K-D, Wang S-S, Hu L-Y, Chen X-Y, Li Y-H, et al (2017) Hydrogen sulfide alleviates postharvest ripening and senescence of banana by antagonizing the effect of ethylene. PLoS ONE 12(6): e0180113 https://doi.org/10.1371/journal.pone.0180113 28662156 10.1371/journal.pone.0180113 PMC5491123

